# Kinematic Model-Based Pedestrian Dead Reckoning for Heading Correction and Lower Body Motion Tracking

**DOI:** 10.3390/s151128129

**Published:** 2015-11-06

**Authors:** Min Su Lee, Hojin Ju, Jin Woo Song, Chan Gook Park

**Affiliations:** 1 Department of Mechanical and Aerospace Engineering, Automation and Systems Research Institute, Seoul National University, Seoul 151-744, Korea; E-Mails: mandu46@snu.ac.kr (M.S.L.); hojin419@snu.ac.kr (H.J.); 2 BK21Plus Transformative Training Program for Creative Mechanical and Aerospace Engineers, Department of Mechanical and Aerospace Engineering, Seoul National University, Seoul 151-744, Korea; E-Mail: jinwoo.song@snu.ac.kr

**Keywords:** indoor positioning, pedestrian dead reckoning, wearable sensors, extended Kalman filter, motion tracking

## Abstract

In this paper, we present a method for finding the enhanced heading and position of pedestrians by fusing the Zero velocity UPdaTe (ZUPT)-based pedestrian dead reckoning (PDR) and the kinematic constraints of the lower human body. ZUPT is a well known algorithm for PDR, and provides a sufficiently accurate position solution for short term periods, but it cannot guarantee a stable and reliable heading because it suffers from magnetic disturbance in determining heading angles, which degrades the overall position accuracy as time passes. The basic idea of the proposed algorithm is integrating the left and right foot positions obtained by ZUPTs with the heading and position information from an IMU mounted on the waist. To integrate this information, a kinematic model of the lower human body, which is calculated by using orientation sensors mounted on both thighs and calves, is adopted. We note that the position of the left and right feet cannot be apart because of the kinematic constraints of the body, so the kinematic model generates new measurements for the waist position. The Extended Kalman Filter (EKF) on the waist data that estimates and corrects error states uses these measurements and magnetic heading measurements, which enhances the heading accuracy. The updated position information is fed into the foot mounted sensors, and reupdate processes are performed to correct the position error of each foot. The proposed update-reupdate technique consequently ensures improved observability of error states and position accuracy. Moreover, the proposed method provides all the information about the lower human body, so that it can be applied more effectively to motion tracking. The effectiveness of the proposed algorithm is verified via experimental results, which show that a 1.25% Return Position Error (RPE) with respect to walking distance is achieved.

## 1. Introduction

The topic of human position tracking has recently attracted a great deal of attention. The ability to locate an individual has resulted in numerous applications, including location-based control, personalized advertisements, evacuation, and first responder’s safety applications, *etc.* The Global Positioning System (GPS) is one of the most powerful positioning techniques used for human applications. It is the most effective solution for positioning in outdoor environments, however, as it suffers signal shielding, the GPS fails to provide a definite device location with sufficient accuracy in an indoor environment. This fact leads to the need for an alternative, accurate and reliable indoor positioning system method. For indoor positioning of a pedestrian, many positioning methods have been proposed using different devices such as a wireless signals, cameras, and inertial sensors.

Most indoor positioning techniques are based on the received signal strength (RSS) of wireless signals, for instance WiFi [1], ultrasound [2], radio frequency identification (RFID) and ultra-wideband (UWB) [3], *etc.* The most commonly applied technique in RSS-based localization is the fingerprinting approach, which requires manual collection of a huge dataset for training purposes [4,5]. Moreover, the fingerprinting approach requires a retraining process whenever the environment is altered. In addition to these problems, the wireless signal-based positioning methods have other fatal disadvantages, such as multi-path problems or the need for significant amounts of *a priori* knowledge. Although signal-based approaches have an advantage for time independent positioning, their use is not appropriate for many other applications such as when entering an unknown building.

Vision-based positioning techniques using cameras has also been intensively investigated as the smartphone market has quickly grown, and the quality of motion capture equipment has improved greatly. Vision-based motion capture systems are already in use for many applications, including the computer graphics of movies and games. Vicon Motion Systems Ltd. (Oxford, UK) provides an example of the commercial development of a vision-based system. Vicon’s technology can deliver the position of each attached marker with reliable accuracy [6]. However, despite the advancements in positioning performance, this device is too expensive and requires a particular environmental infrastructure. Image matching that uses feature extraction can be a promising alternative solution for large-scale environments [7]. With this technique, images which are captured by the localization system are matched with reference images based on features. Then, for a location estimation, the known location of the most similar reference image is used. Scale and rotation-invariant features [8] makes the matching process work well even if the reference image was captured from a different angle or position.

Pedestrian positioning using inertial sensors has been employed in a wide range of fields including ambulatory human movement analysis. Micromachined gyroscopes and accelerometers are used in several applications, which include monitoring of daily living activities [9,10,11], assessment of internal mechanical working load in ergonomics studies [12,13,14,15], measurement of neurological disorders [16,17,18,19], and mixed and augmented reality [20,21,22]. Tracking the location of a human using an inertial sensor has always been a very important problem and a great challenge for many indoor and outdoor applications because the inherent drift of the orientation and position estimates restricts long-term stable use of these sensors [23]. One of the most reliable and stable algorithms among many studies on human positioning is the Pedestrian Dead Reckoning (PDR), a type of personal navigation. It employs inertial sensors to gather the “step information” or distinct characteristics from a pedestrian. This is used to analyze his or her specific walking behavior and calculate the total walking distance. The PDR can be utilized under various circumstances, as it is autonomous and insusceptible to external jamming. Thus far, the major application of the PDR has been to predict the next movement of its user and to precisely calculate his or her total walking distance using the acquired step information [24]. According to the position of the installed sensors, the PDR can be categorized as foot-mounted [25,26,27,28], waist-mounted [29,30], and handheld [31,32,33] types. Handheld type PDR usually refers to a smartphone application. In this case context awareness of the person is another issue for pedestrian positioning since the dynamic context affects the sensor attitude accuracy. Smartphone-based PDR considering pedestrian’s speed [34] or context awareness [35,36] have been studied. Fusion PDR with WiFi signals or environment information is also an important part of pedestrian navigation [37,38]. PDR can also be divided into two types, foot mounted sensor (INS) and Step and Heading System (SHS, for other mounted sensors) which also refer to Harle’s work [24]. Unlike the other type, PDR using foot-mounted sensors is similar to a strap down system. Basically it follows the Inertial Navigation System (INS) and is corrected using the characteristics of the foot during walk. In this paper, we uses the word PDR for an algorithm using a foot-mounted sensor. Using the foot-mounted inertial sensors, the Zero velocity UPdaTe (ZUPT) algorithm provides the most powerful step length estimation results. The ZUPT reduces the most significant amount of Inertial Measurement Unit (IMU) system errors that are caused by the bias drift of the accelerometers. When a pedestrian walks, a stance phase occurs and at that moment, the velocity of the foot is practically zero. The ZUPT uses this stance moment and makes the velocity of the IMU zero, which an acceleration integral does not achieve. A disadvantage of the ZUPT is that it cannot solve heading problems. The heading of the pedestrian easily diverges due to the bias of the gyro, such as magnetometer disturbance and sensor-to-foot misalignment [39]. To solve the heading problem, Zero Angular Rate Update (ZARU) [40], Magnetic Angular Rate Update (MARU) [41] and Heuristic Drift Elimination (HDE) [42] algorithms are suggested. Similar studies using multiple sensors for human motion tracking and positioning are introduced in [43,44,45]. However, Xsens MVN systems were not able to solve the positioning problem and Simultaneous Localization And Capture (SLAC) did not include the yaw drift error. For the dual-foot system, the foot heading cannot be used to determine the actual heading of the pedestrian because each foot delivers different headings.

This study presents an enhanced method for finding the heading and position of a pedestrian by using a kinematic equation with data from wearable sensor measurements. Seven inertial sensors are attached at each segment of the lower body to track attitudes. In the proposed algorithm, the heading of the pedestrian is estimated through the inertial sensor that is attached at the waist. Unlike other segments, the waist sensor employs a magnetometer. In classical PDR the walking direction was typically estimated by means of the magnetometer as this can be quite effective when the device is far from ferromagnetic objects (and more, in general, far from deviations of the magnetic field). In general, the magnetic norm of the each foot is over one and has a large variance in indoor and outdoor environments. Unlike the foot, a waist sensor is located far from the ground and attached to a position with little movement that it shows a stable magnetic norm. The Extended Kalman Filter (EKF) is used for the integration of the heading that is estimated by the magnetometer, position and velocity of the waist, which are all estimated through the kinematic model. Then, the estimated waist position and heading are used to calculate the position of the other segments. Through the proposed algorithm, the accurate position of waist can be acquired with the ZUPT, and each leg can yield physically related positions using the waist position. The proposed algorithm can be applied for various fields, such as positioning of pedestrians including first responders, 3D human motion capture, and rehabilitation training.

The reminder of this paper is organized as follows: in Section 2, the system of the proposed algorithm is introduced with the aid of a flowchart. An algorithmic summary of the approach will be explained. Section 3 introduces the detailed operations of the proposed algorithm. In this section foot positioning using the ZUPT is introduced. The use of the EKF is explained, and the limitations of the ZUPT are also discussed in this section. This section also describes calibration, the Attitude Reference System (ARS), EKF for waist localization, and re-estimation of each segment. Section 4 presents the verification tests for the proposed algorithm and discusses the results in comparison with vision-based positioning. Finally, Section 5 concludes the study with a summary.

## 2. System Overview

**Figure 1:**
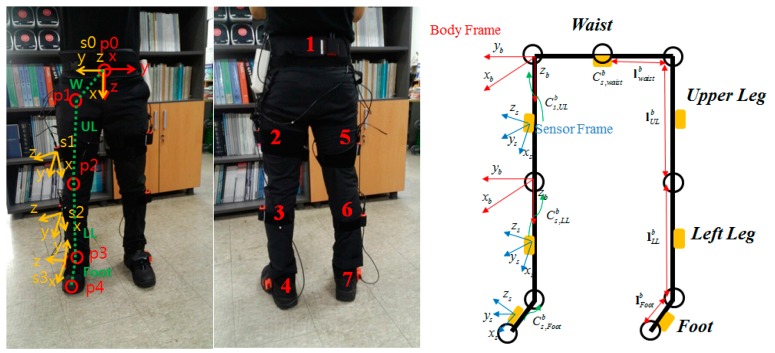
Location of attached sensors and kinematic model.

Figure 1 shows the front and back of the subject with the seven sensors mounted on the body. The sensor outputs are wirelessly sent to the PC through USB receivers and Bluetooth modules. The Xsens XBUS systems are used for the IMU device and logger, which receives simultaneous data from every sensor. Each of these sensor modules includes a MEMS grade tri-axial accelerometer, a tri-axial gyroscope, and a tri-axial magnetometer. The system uses the magnetometer only for estimation of heading with the waist-mounted sensor because any disturbance in the magnetometer can greatly affect the other positions. The seven inertial sensors are attached to the feet, lower legs, upper legs, and the center of the waist. Each sensor is tightly wrapped in order to minimize any skin motion effects. The length of each joint is previously measured for the kinematic relation calculation. The start and end points of the segments are located at the center of the joint as illustrated in Figure 1. The sensor and body frames are defined as follows: the red arrows represent the definition of the body frame. The z-axis points to the center of the Earth, and the x-axis points the front of the subject. All joints use the same axis frames. The yellow arrows show the sensor frames. A kinematic model is used to calculate the position and velocity of waist rooted from the foot position. Detailed equations for the kinematic model will be described with Equations (13) and (14). After calculating the position of the waist, the position of the feet could be calculated using a kinematic model again like Equation (18).

**Figure 2:**
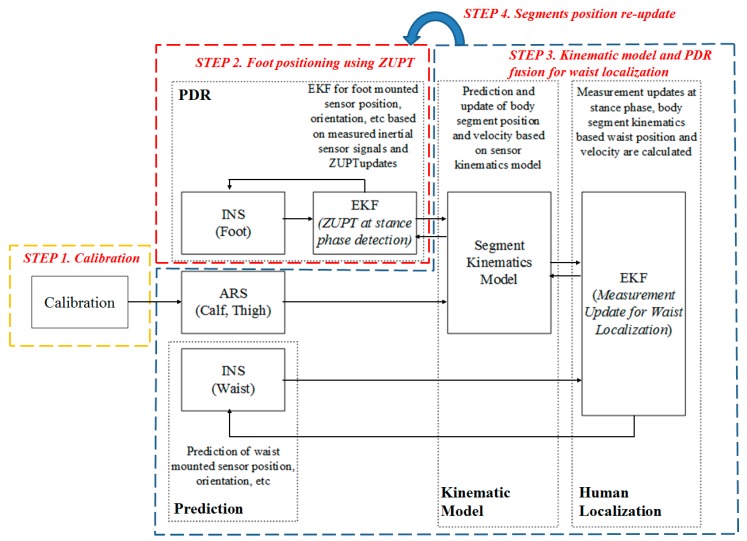
Block diagram of the kinematic model and PDR fusion algorithm.

The block diagram of the proposed algorithm is shown in Figure 2. When the sensor signals are measured, each sensor undergoes different processes according to the attached position. Attitude and position of the feet and waist mounted sensor are calculated using Inertial Navigation System (INS) and corrected by EKF. At the upper and lower leg segments (thigh and calf), only the orientation is calculated using the Attitude Reference System (ARS) with the measured inertial sensor signals. The filter structure that is used to reduce sensor errors is difficult to apply to these segments as the position of the sensor is difficult to calculate. The positions and velocities of these segments are calculated using the kinematic model to reduce processing time. By using a known sensor to body alignment and a kinematic model of the body, prediction of the segment kinematics could followed. The sensor noise, sensor signal offset, or sensor orientation errors lead to a drift error when integrating inertial sensor data over time. Estimated velocity, position and orientation are continuously updated in the sensor fusion scheme to correct the estimated quantities. The correction step includes updates based on kinematic relationship of the human body when the stance phase is detection. The ZUPT algorithm is adjusted to each foot sensor, which yields a zero velocity measurement during the stance phase. Using the foot position of each leg, the position and velocity of waist can be calculated using the kinematic model. These measurements and the magnetometer-based heading are used to reduce the waist-mounted sensor error. The estimated waist position and heading are fed back to each segment using the backward kinematic model, and the feet positions are updated to be used in the next time frame.

The summary of the proposed algorithm can be explained with four steps as shown in Figure 2. First, a calibration process is required. In the calibration, misalignment of the calf and thigh sensor are calculated. The second step of the algorithm is foot positioning using ZUPT. When the stance phase is detected, zero velocity measurements are updated during the phase. Step 3 is the waist localization part which is calculated by means of kinematic models. Position, orientation and sensor errors of the waist attached sensor are estimated with the measured position and velocity of the waist inferred from the foot position using the kinematic model. In order to use the kinematic model, the orientations of leg segments are required and estimated with a typical ARS algorithm and the results of the calibration step. Step 4 re-updates the segment positions. The positions of calf, thigh and foot are calculated with the kinematic model, and the EKF for the foot mounted sensor uses the corrected foot position measurement. Detailed operation of each step will introduced in Section 3.

## 3. EKF-Based PDR and Kinematic Model Fusion

### 3.1. Calibration

Before adjusting the algorithm, calibration processes are required to identify several kinematic parameters of the limbs. The rotation matrix between the sensor frames and the limb body frames are calculated so that the sensor data collected in the sensor frames can be accurately transformed into data in the limb body frames. In other words, the purpose of the calibration process is to acquire the rotation from the sensor to body segment, $C_{s}^{b}$. To find the sensor to segment alignment, the subject is asked to stand in a previously defined pose. The rotation from sensor to body segment, $C_{s}^{b}$, is determined by matching the orientation of the sensor in the global frame $C_{s}^{n}$ to the known orientation of each segment $C_{b}^{n}$ (Equation (1)):

(1) $$C_{b}^{n}=C_{s}^{n}\cdot C_{b}^{s}$$

For calibration, the measured accelerometer is used to get roll and pitch. The roll and pitch rotation can be calculated during the standstill phase using gravity force (Equations (2) and (3)), where $f_{x}^{s},f_{y}^{s}\;\text{and}\;f_{z}^{s}$ are the accelerometer frames with respect to the sensor frame. The definitions of the sensor frames follow the depiction in Figure 1.

(2) $$\phi (0)={\text{tan}}^{-1}\left(\frac{f_{y}^{s}}{f_{z}^{s}}\right)$$

(3) $$\theta (0)=-{\text{tan}}^{-1}\left(\frac{f_{x}^{s}}{f_{z}^{s}}\right)$$

**Figure 3:**
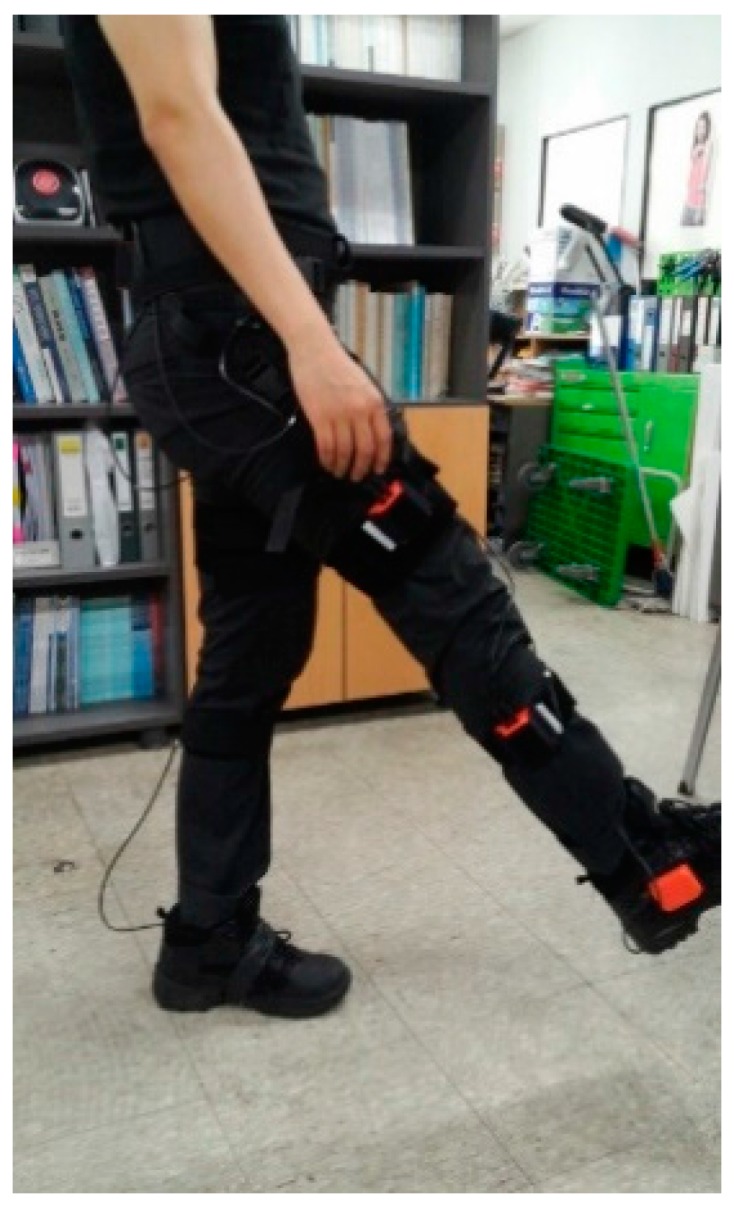
Lifting the right leg for the calibration process. The subjects is asked to perform the motion three times each for both legs.

However, as the yaw rotation cannot be detected using the gravity force, so the quaternion update method is used as well. In this study, the subject is asked to stand still for a few seconds, lift up his or her leg without bending the knee, and stand still again (Figure 3). This process is repeated three times to get an averaged value. Identical processes are performed for the right and left legs. During this process, we assume that the lifting leg posture rotates around only the y-axis of the body frame. Therefore, gyro signals during the lifting posture are supposed to have changed only in the y-axis gyro. Misalignment of the frame induces gyro signals at other axes. Therefore, this signal assists in detecting the rotation of the yaw angle.

**Figure 4:**
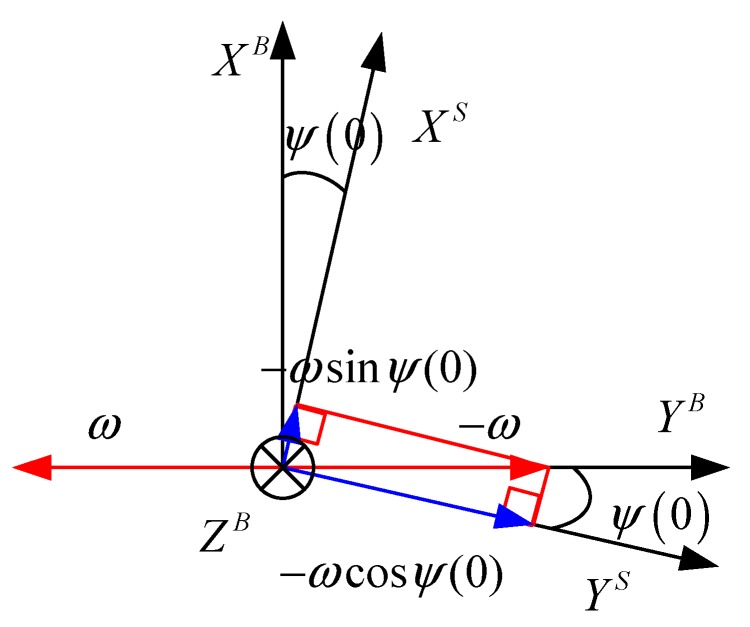
Misalignment of yaw effects gyro signals at other axis during Y-axis rotation.

Figure 4 shows the effect of misalignment on the gyro signals that Equation (4) employs to calculate yaw:

(4) $$\psi (0)={\text{tan}}^{-1}\left(\frac{\omega_{x}^{s}}{\omega_{y}^{s}}\right)$$

**Figure 5:**
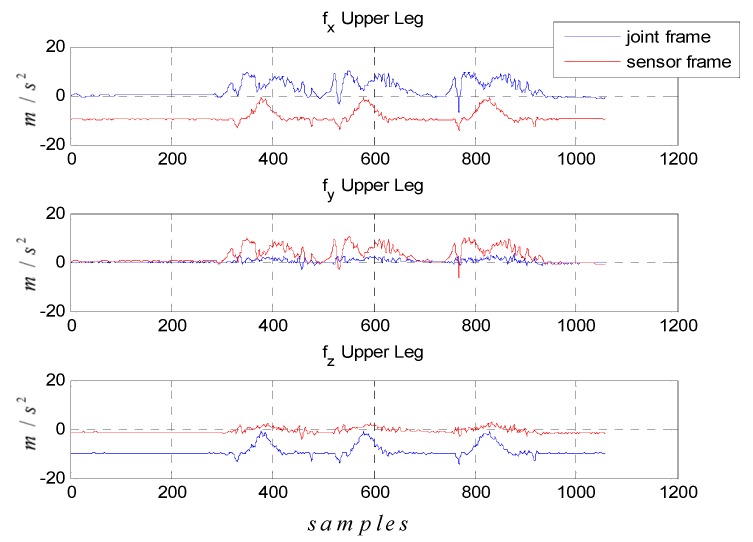
Accelerometer signals of upper leg during calibration motion which represented at sensor frame (**red line**) and joint frame (**blue line**).

Figure 5 shows the time histories of the accelerometers on the upper legs during the lifting motion. The red lines and blue lines indicate the accelerometer reading with respect to the sensor frame and the body frame, *i.e.*, at the joint, respectively. When the subjects remain in the standstill phase for the first ten seconds in the graph, the accelerometer in the body frame is supposed to experience gravity force on the z-axis. Furthermore, the result of Figure 5 indicates that the rotation matrixes are correctly acquired.

### 3.2. Foot Positioning Using ZUPT

Recently, tracking the position of a pedestrian using a foot-mounted IMU has been actively researched. Among the available methods, this study implemented the INS-EKF-ZUPT (IEZ) algorithm [46], which reduces the drift of the sensor with zero velocity measurements at the stance phase of the foot. Because the ZUPT assumes that velocity of the foot is zero at the stance phase, this measurement propagates to the INS-EKF. Although this algorithm has an advantage as it is easy to implement and provides accurate step length estimation, the IEZ algorithm requires *a priori* accurate stance phase detection.

The gait can be simply divided into the stance phase and swing phase. The stance phase represents a section of the contact between the shoes and the ground. Most algorithms in the literature generally determine the stance phase based on gyroscope and accelerometer signals. However, methods that simply use sensor signals and classify walking status by the threshold suffer problems of robustness for various motions. In order to correctly detect the stance phase, this study employs modified signals and a combination of them, which show significant differences for all motions such as slow walking, crawling and random walking [47].

**Figure 6:**
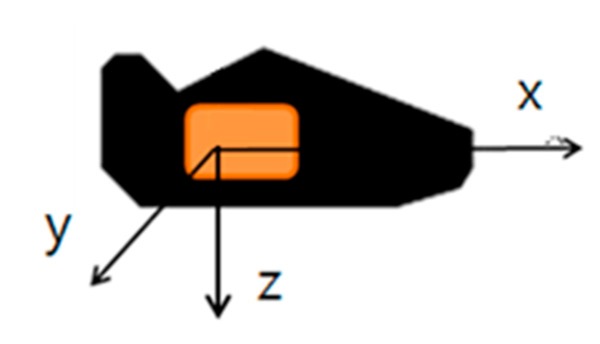
Frame of the sensor attached at the right foot.

**Figure 7:**
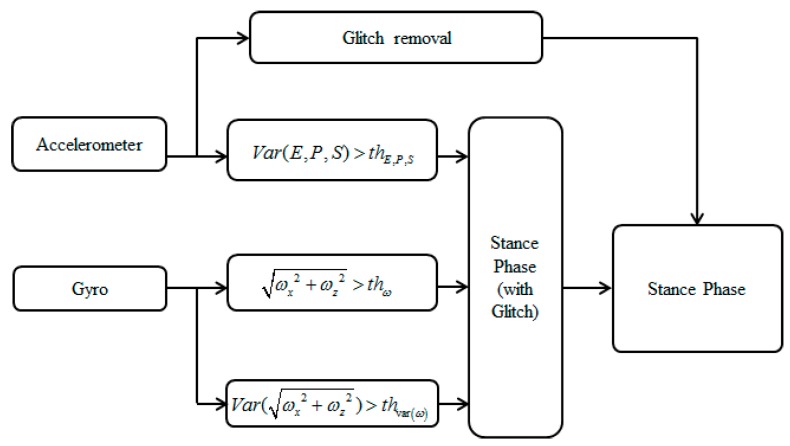
Block diagram of the stance-phase detection algorithm.

The definition of the sensor frame of reference that is attached on the right foot is depicted in Figure 6. The x-axis is pointing forward, and the z-axis is directed towards the ground. Figure 7 shows a block diagram of the step phase detection procedure. In this study, the stance phase is detected simply using modified signal features and glitch removal. The x and z-axis accelerometer output values are used, which are altered significantly when the pedestrian is in motion. The procedure is as follows: first, to detect the stance phase, three modified accelerometer signals are used: the energy, product, and sum. As given in Equation (5), the energy is the root square sum of the x and z-axis accelerometers outputs; the product denotes their multiplication, and sum is their summation. Next, local variances of energy, product, and sum are computed. If each local variance is below a threshold, then that specific condition is marked as 1 which means situation satisfied the condition, as shown in Equation (6). However, because the three conditions use only x and z-axis accelerometers outputs, the stance-phase algorithm cannot be engaged for side walking, crawling, descending stairs, ascending stairs, and other types of movements:

(5) $$\begin{array}{l} Energy=\sqrt{{a_{x}}^{2}+{a_{z}}^{2}}\\ \text{Pr}oduct=a_{x}\cdot a_{z}\\ Sum=a_{x}+a_{z} \end{array}$$

(6) $$\begin{array}{l} {Condition}_{E}=\left\{ \begin{array}{cc} 1 & \text{var}(E\left(k-14\right):E\left(k\right))<{th}_{E}\\ 0 & otherwise \end{array}\right.\\ {Condition}_{P}=\left\{ \begin{array}{cc} 1 & \text{var}(P\left(k-14\right):P\left(k\right))<{th}_{P}\\ 0 & otherwise \end{array}\right.\\ {Condition}_{S}=\left\{ \begin{array}{cc} 1 & \text{var}(S\left(k-14\right):S\left(k\right))<{th}_{S}\\ 0 & otherwise \end{array}\right. \end{array}$$

In order to improve the performance of the stance-phase detection, this study supplements the accelerometer outputs with the gyroscope outputs. As shown in Equations (7) and (8), the condition is determined by the root square sum of the x- and y-axis gyroscope outputs. If $\left|w_{k}\right|$ is below a threshold, the condition is marked as 1, as shown in Equation (7). If the local variance of $w_{k}$ is below a threshold, the condition is marked as 1, as shown in Equation (8).

(7) $$\begin{array}{l} \omega \left(k\right)=\sqrt{{\omega^{2}}_{x}\left(k\right)+{\omega^{2}}_{y}\left(k\right)}\\ {Condition}_{\omega }=\left\{ \begin{array}{cc} 1 & \left|\omega \left(k\right)\right|<{th}_{\omega }\\ 0 & otherwise \end{array}\right. \end{array}$$

(8) $${Condition}_{\text{var}.\omega }=\left\{ \begin{array}{cc} 1 & \text{var}(\omega \left(k-14\right):\omega \left(k\right))<{th}_{\text{var}(\omega )}\\ 0 & otherwise \end{array}\right.$$

**Figure 8:**
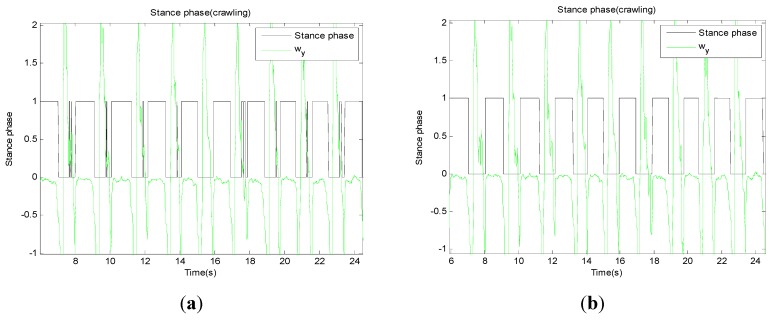
Result of the stance phase detection. Green line shows y-axis gyro and black line shows phase condition. (**a**) shows result of stance phase detection without glitch removal and (**b**) shows without it.

If all conditions are simultaneously flagged as 1, that point can be considered as a stance phase. However, this is not enough to detect the accurate stance phase because of the limitations of using a threshold. Even if the swing phase is incorrectly detected during the stance phase, this causes only a slight problem because the actual moving distance is nearly zero. However, if it happens during the swing phase, this causes a major problem because the actual velocity may be significant. Therefore, in order to eliminate false detections steps during the swing phase, this study employs a glitch removal process. If the duration of the phase change is too short, the system ignores the phase change and retains the previous phase. Figure 8 shows an example of the stance phase detection during normal walking. The black line goes to 1 when the stance phase is detected, and some fault detections are eliminated through the glitch removal algorithm. A combination of the modified accelerometer and gyro signals at the foot-mounted IMU can detect the stance phase in various motions within the miss detection rate of 2% [48].

**Figure 9:**
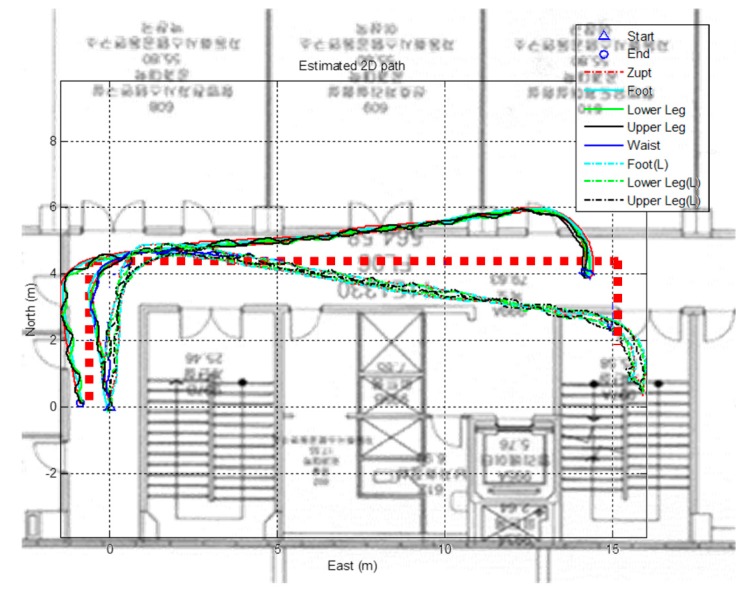
Result of PDR using foot-mounted IMU applied at dual foot. The solid line is position of right leg segments and the dotted line is position of left leg segments.

During the stance phase, the IEZ delivers the zero velocity measurements to the EKF model. The proposed algorithm considers a state vector formed by 15 variables, *i.e.*, accelerometer bias Equation (3), gyro bias Equation (3), attitude error Equation (3), velocity error Equation (3), and position error Equation (3), and employs zero velocity at the stance phase for measurements as shown in Equations (9)–(12):

(9) $$\delta x={\left[{\delta \varphi }^{T},{\delta b}^{\omega^{T}},{\delta p}^{T},{\delta v}^{T},{\delta b}^{a^{T}}\right]}^{T}$$

(10) $$\begin{array}{l} \delta \varphi \left(k+1\right)=\delta \varphi \left(k\right)+{\hat{C}}_{b}^{n}\left(k\right){\delta b}^{\omega }\left(k\right)\Delta t\\ {\delta b}^{\omega }\left(k+1\right)={\delta b}^{\omega }\left(k\right)\\ \delta p\left(k+1\right)=\delta p\left(k\right)+\delta v\left(k\right)\Delta t\\ \delta v\left(k+1\right)=\delta v\left(k\right)-({\hat{b}}^{a}\left(k\right)\times )\delta \varphi \left(k\right)\Delta t+{\hat{C}}_{b}^{n}\left(k\right){\delta b}^{a}\left(k\right)\Delta t\\ {\delta b}^{a}\left(k+1\right)={\delta b}^{a}\left(k\right) \end{array}$$

(11) $$H=\left[\begin{array}{ccccc} 0_{3\times 3} & 0_{3\times 3} & 0_{3\times 3} & I_{3\times 3} & 0_{3\times 3} \end{array}\right]$$

(12) $$z={\left[\begin{array}{ccc} 0 & 0 & 0 \end{array}\right]}^{T}$$

where, δ**φ** is the attitude error, ${\delta b}^{\omega }$ is the gyro bias, δ**p** is the position error, δ**v** is the velocity error, ${\delta b}^{a}$ is the accelerometer bias, **H** is the measurement model, and **z** is the velocity measurement at the stance phase. Equation (10) represents the system model equation. This system provides a relatively accurate position of the pedestrian both indoors and outdoors. It is reported that generally the positioning error of IEZ with closed-loop trajectories is over 15% (125 m walk) Return Position Error (RPE). Since this method does not have yaw direction observations, the gyro drift is poorly estimated. For example, when implementing the IEZ systems on a dual foot system, each foot yields different positions of the pedestrian due to gyro bias or sensor misalignment. Figure 9 shows the test results of the IEZ algorithm applied to the dual foot scenario.

The red dotted line is the actual trajectory. The solid lines represent the position of the right leg segment, which updates using the kinematic model rooted on the right foot position, and dotted lines represent the position of the left leg. The initial heading of each leg is provided, and the trajectory is updated according to the measured gyro signals. The subjects are asked to walk along the actual trajectory lines and return to the starting position. The two algorithms deliver two distinct directional trajectories although the end positions are close to the starting point. In this case, it is not possible to conclude on which trajectory the real position of the pedestrian is. Therefore, this study proposes integrating a kinematic model with the PDR using multiple wearable sensors, which enables one to precisely locate the pedestrian.

### 3.3. Kinematic Model and PDR Fusion for Waist Localization

**Figure 10:**
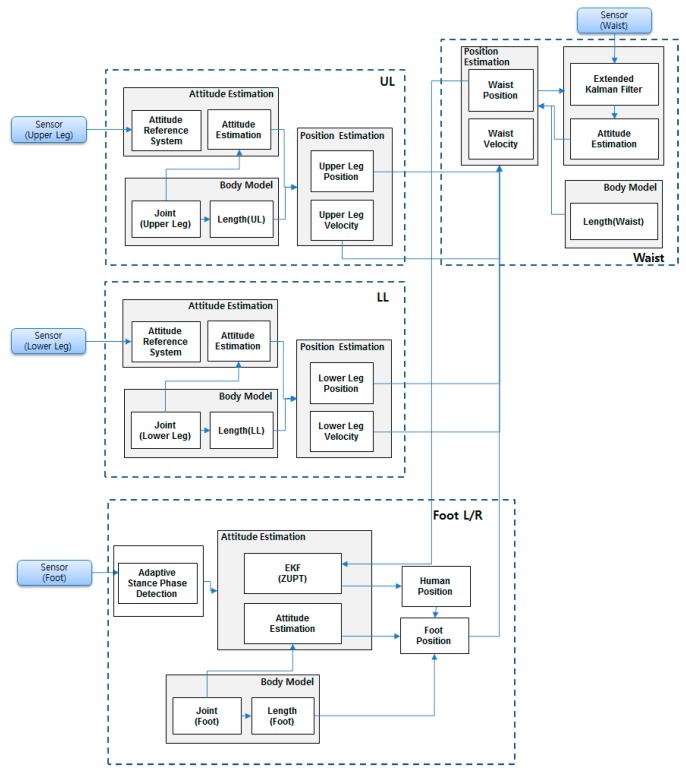
Flowchart of kinematic model and PDR fusion algorithm.

To reduce the error of the waist-mounted sensor, the EKF is adjusted using the three types of measurements: the magnetometer-based heading, position and velocity of the waist, which are calculated using the kinematic model. Figure 10 shows a flowchart of the proposed algorithm. The orientations of the segments are calculated using the ARS algorithm with the measured upper and lower leg-mounted sensor signals. The orientation and position of the dual foot are estimated from the PDR algorithm that was provided in the previous the section. 

To calculate the attitudes of lower and upper legs, the adaptive ARS is implemented at each segment. These segments are subject to dynamic movements when the pedestrian is walking. Therefore, the heading estimation can be unstable, and disturbance of magnetometer increases as it nears the ground. Therefore, this study selects the ARS rather than Attitude Heading Reference System (AHRS). The Gauss-Newton method is used for the ARS. In the adaptive ARS algorithm, the roll, pitch and yaw of the sensor are calculated using the accelerometer and gyro. However, as the yaw is only calculated from the gyro update, the results that the system yields can be unreliable. In order to enhance the reliability of the system, the measurement covariance *R* adaption method is implemented. When the subject’s motion is stable, the *R* value is small, and the attitude from the accelerometer becomes a reliable value. In contrast, when the subject’s motion is dynamic, the *R* value is larger so that the attitude from the gyro becomes a reliable value. Through this process, it is possible to derive the attitude of the sensor with respect to the navigation frame, $C_{s}^{n}$. Combining this result with the sensor-to-body relationship, which is calculated from the calibration process, results in attitude of the body in the navigation frame, $C_{b}^{n}$, as shown in Equation (9). The attitude of each segment is then used for the kinematic model computation.

The waist is located at the center of the human body. The pure INS results, which use only the waist-mounted sensor, show divergent orientation and position due to the sensor errors. To reduce the errors, a filter that carries measurements with physical meaning is required. This study proposes the EKF-based waist position estimation algorithm using measurements from the two legs. Using the biomechanical model, the position and velocity of the waist can be calculated during the stance phase of each leg. The heading of the waist is corrected using the magnetometer-based heading computation.

**Figure 11:**
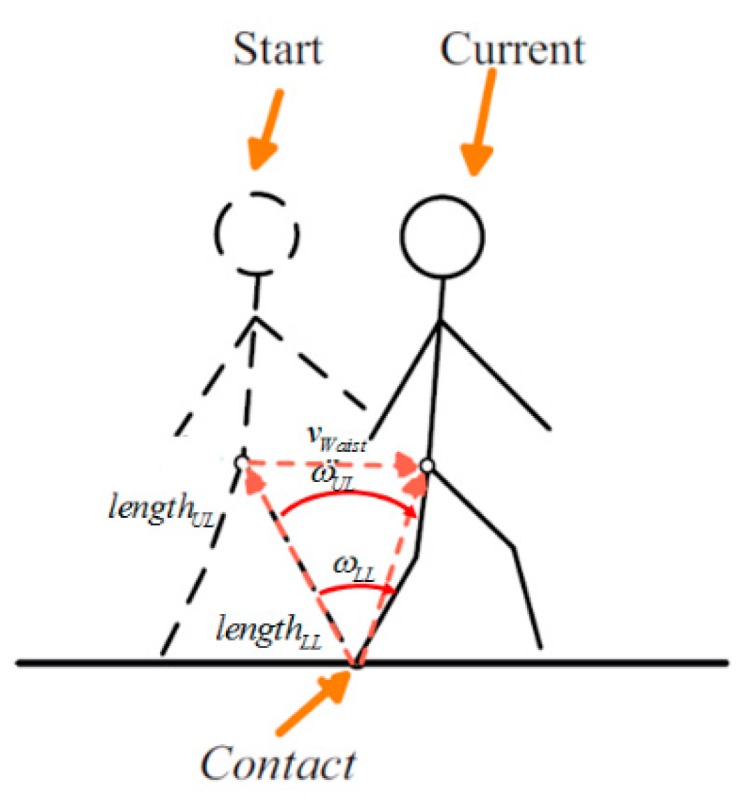
Movement of waist could be assumed to pivot motion when a foot is connected to ground.

Velocity of the waist is calculated at stance phase using the biomechanical model. Figure 11 shows the concept of the velocity model. During the stance phase, movement of the leg creates a pivot motion with the center of the shoe. In between the shoe and the waist, there are three segments, *i.e.*, lower leg, upper leg, and waist, and the velocity of the waist is computed as the sum of the three pivot movements. Equation (13) describes the kinematic model of velocity measurement and is applied to the left and right legs:

(13) $$v_{waist}^{kinematic\;\text{mod}el}=C_{b,LL}^{n}\left(C_{s,LL}^{b}\omega_{LL}^{s}\times l_{LL}^{b}\right)+C_{b,UL}^{n}\left(C_{s,UL}^{b}\omega_{UL}^{s}\times l_{UL}^{b}\right)+C_{b,Waist}^{n}\left(C_{s,Waist}^{b}\omega_{Waist}^{s}\times l_{Waist}^{b}\right)$$

$v_{waist}^{kinematic\;model}$ represents velocity vector of waist measured by kinematic model, $C_{b,segment}^{n}$ is attitude of body at each segment (LL: Lower Leg, UL: Upper Leg and Waist) in navigation frame. $C_{s,segment}^{n}$ is attitude of sensors at each segment in navigation frame. $\omega_{segment}^{s}$ is gyro signal of each segment represented in sensor frame. $l_{segment}^{b}$ is length vector of each segment represented in body frame.

The INS kinematics is converted to the body segment kinematics using a biomechanical model. This model assumes that a subject’s body includes body segments linked by joints and the sensors are attached to the subject’s body segments. Joint origins are determined by the anatomical frame and are defined in the center of the functional axes with directions x, y and z being related to functional movements. In the proposed algorithm, the position of foot, which is computed from the IEZ algorithm, is the root position of the kinematic update. The position of the waist using the kinematic model can be calculated through Equation (14).

(14) $$p_{waist}^{kinematic\;\text{mod}el}=p_{Foot}^{EKF}+C_{s,LL}^{n}C_{b,LL}^{s}l_{LL}^{b}+C_{s,UL}^{n}C_{b,UL}^{s}l_{UL}^{b}\;+\;C_{s,Waist}^{n}C_{b,Waist}^{s}l_{Waist}^{b}$$

$p_{waist}^{kinematic\;model}$ represents position vector of waist measured by kinematic model. $p_{Foot}^{EKF}$ is position vector of foot corrected from EKF for foot and $C_{b,segment}^{s}$ is rotation matrix body to sensor frame.

Similar to other skin-based systems, skin and soft tissue artifacts influence the measurements of the sensors due to contracting muscles, skin, and fat. Furthermore, the error of attitude at each segment is one of the most significant sources of position error. As a result, although the position or orientation changes of the segments around a joint can be measured, they seldom represent biomechanically true values. In the joint measurement update step, these artifacts are reduced using the average, while this needs to account for the statistical uncertainties.

For the waist sensor, the same 15-state linearized function as the EKF is used for the foot-mounted IMU, which is formulated as Equation (5). The system model of the waist-mounted IMU is also identical to the filter for the foot-mounted IMU shown in Equation (6).

Measurement is updated at the stance phase of each foot. In other words, when the right foot is on the stance phase, the waist position and velocity estimated from right leg using the kinematic update calculation are used. The same procedure is conducted for the left leg during its stance phase. 

The heading from the magnetometer implemented at the waist-mounted IMU is also used for measurements. The other measurements, including the orientation of the dual foot, do not deliver yaw orientation that is related to the true north direction. This measurement corrects the heading of the pedestrian using Equation (15).

(15) $$\psi ={\text{tan}}^{-1}\left(\frac{-Y_{m}\text{cos}\phi +Z_{m}\text{sin}\phi }{X_{m}\text{cos}\theta +Y_{m}\text{sin}\theta \text{sin}\phi +Z_{m}\text{sin}\theta \text{sin}\phi }\right)$$

Therefore, the measurement model equation *H* is formulated as shown in Equation (16). The measurement vector **y** is formulated as shown in Equation (17)

(16) $$H=\left[\begin{array}{ccccc} 0_{3\times 3} & 0_{3\times 3} & {Ι}_{3\times 3} & 0_{3\times 3} & 0_{3\times 3}\\ 0_{3\times 3} & 0_{3\times 3} & 0_{3\times 3} & {Ι}_{3\times 3} & 0_{3\times 3}\\ \left[0\;0\;1\right] & 0_{1\times 3} & 0_{1\times 3} & 0_{1\times 3} & 0_{1\times 3} \end{array}\right]$$

(17) $$y_{k}=\left[\begin{array}{c} p_{k|k-1}-p_{waist,k}^{kinematic\;\text{mod}el}\\ v_{k|k-1}-v_{waist,k}^{kinematic\;\text{mod}el}\\ \psi_{k|k-1}-magnetic\;yaw \end{array}\right]$$

where, $p_{k|k-1}$ represents the estimated position, $v_{k|k-1}$ represents the estimated velocity, and $\psi_{k|k-1}$ represents the estimated yaw.

Noises of measurement and systems w and v are predictable from the sensor specifications and physical consideration. For example, covariance error of the position of the waist set to 1m, by the means of empirical manner.

### 3.4. Segments Position Re-Update

The previous algorithm steps may not be sufficient to estimate the segment positions because the positions of the leg models are not associated with each other, and yield different headings from that of the foot. In order to relieve this limitation, the proposed method employs one more step in which each segment establishes a physical connection with the estimated waist position. With the estimated position of the waist, the position of segments can be calculated using the inverse kinematic model rooted from the waist position. The procedure is as follows: The system first re-calculates the positions of the upper and lower legs using the inverse kinematic model shown in Equation (18):

(18) $$p_{Foot}^{kinematic\;\text{mod}el}=p_{waist}^{EKF}-C_{s,Waist}^{n}C_{b,Waist}^{s}l_{Waist}^{b}\;-C_{s,UL}^{n}C_{b,UL}^{s}l_{UL}^{b}-C_{s,LL}^{n}C_{b,LL}^{s}l_{LL}^{b}$$

$p_{Foot}^{kinematic\;model}$ is the position vector of foot calculated by kinematic model and $p_{waist}^{EKF}$ is position vector of waist corrected by EKF for waist sensor.

The position of the dual foot is continuously corrected according to the measured foot position from the kinematic model. Then, the filter is updated using the EKF for the foot-mounted IMU. Equation (19) shows the measurement of foot position error and Equation (20) shows measurement update matrix for re-updating:

(19) $$y_{k}^{Foot}=\left[p_{k|k-1}^{Foot}-p_{Foot,k}^{kinematic\;\text{mod}el}\right]$$

(20) $$H=\left[\begin{array}{ccccc} 0_{3\times 3} & 0_{3\times 3} & {Ι}_{3\times 3} & 0_{3\times 3} & 0_{3\times 3} \end{array}\right]$$

where, $p_{k|k-1}^{Foot}$ represents the estimated position of foot, $p_{Foot,k}^{kinematic\;model}$ is position vector of foot measured by kinematic model at k epoch. 

By correcting the position of the feet, the position of left and right foot cannot be apart because of the kinematic constraints of the body. For the next time step, the algorithm can provide more accurate measurements of the position and velocity of the waist.

## 4. Experimental Results

In order to verify the accuracy of the positioning system, various walking trajectories are tested with five randomly selected subjects who perform various physical movements. All the subjects undergo the calibration process equally before the walking trajectory tests. The origin point is established as the initial position, and the initial heading of all segments are aligned with the heading of the waist, which is measured using the magnetometer. One performance indicator, RPE, is used when reference data is difficult to obtain. Subjects walk in a closed-loop and return to the initial position. Then, the distance from the final position to the initial position with respect to total walking distance shows the performance of algorithm. RPE does not show drift errors or heading errors effectively. However, in an indoor experimental environment, reference data is difficult to acquire, so the performance index, RPE, is usually used by comparing with real map data.

**Figure 12:**
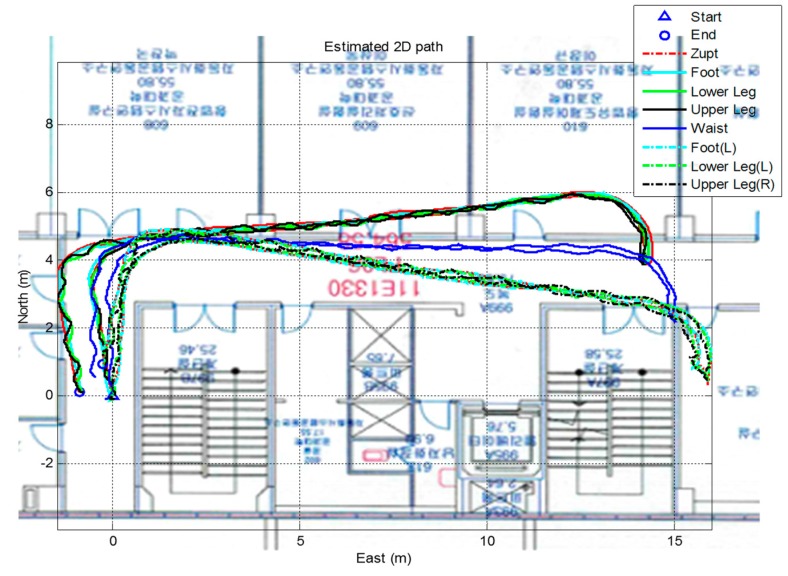
Result of the proposed algorithm compared with feet position using the ZUPT algorithm.

The first test is conducted in an indoor environment. This test provides verification of the performance through comparison with the true trajectory. The 80 m-long closed loop trajectory is tested in the corridor of the building. Unlike Figure 4 which shows the significant heading errors of the ZUPT algorithm, Figure 12 indicates that the proposed algorithm, shown in the blue line, successfully follows the true trajectory, shown in the red line. The RPE of the proposed algorithm is 0.03 m during the 80 m-walk. 

**Figure 13:**
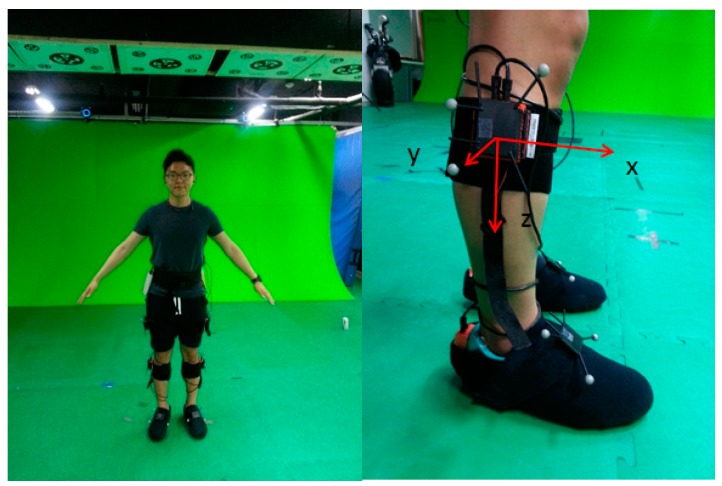
Experimental setting to compare the results of the proposed algorithm with the Vicon setup.

**Figure 14:**
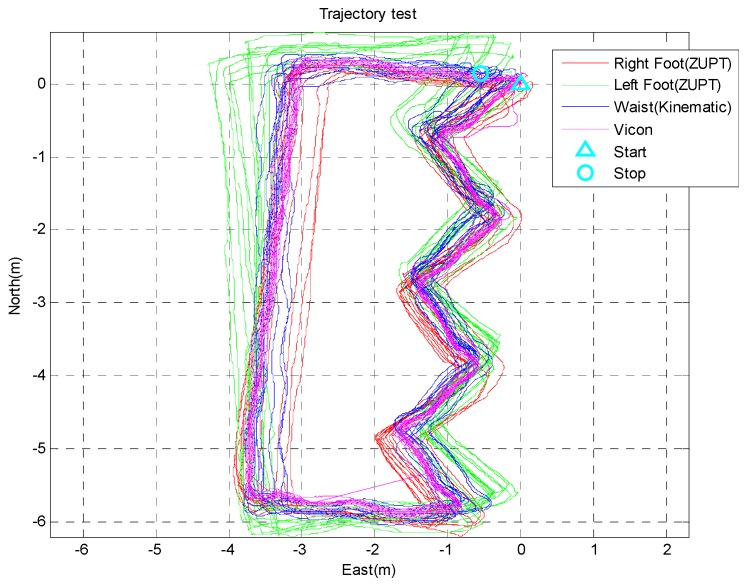
Results of the proposed algorithm and the Vicon system for a 230 m walk.

**Figure 15:**
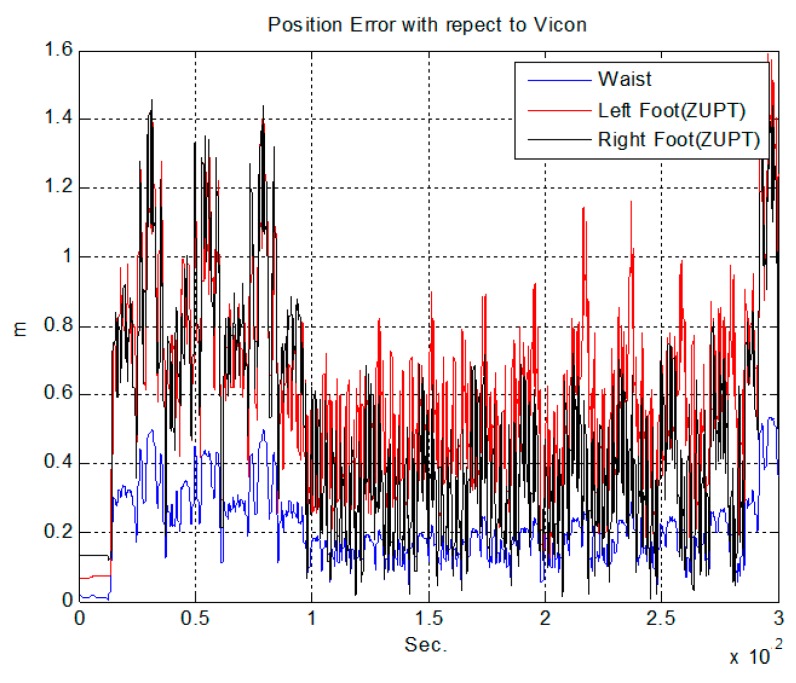
Position error of the proposed algorithm and ZUPT algorithm with respect to the Vicon data.

Another verification test is a comparison with a vision-based positioning system, which is known to provide very accurate positioning (less than 0.1 m) in a restricted area. The true trajectory of this test is a closed loop with a zig-zag line. The subjects walk this loop for five minutes resulting in a total distance of 280 m. Figure 13 shows the system settings of the experiment. The vision system consists of nine Vicon cameras in a 10 m × 7 m area. The Vicon markers are attached at each segment sensors like the right of Figure 13 and fixed to the human body. The Vicon program gives the position information of all the segments with high accuracy. Figure 14 shows the results of the indoor experiment with different algorithms. The magenta line is position of the waist measured by the Vicon setup. As shown in the figure, subjects were asked to walk a closed loop in a zig-zag line. The red and green lines show the results of the algorithm without updating the segment position rooted at the waist. As previously explained, distinctive trajectories are developed for the position of each leg. In contrast, the waist position of the proposed algorithm (blue line) shows a similar line compared with the Vicon line. The two results show similar paths, and the average error of all the subjects’ positions compared to the vision-based system is 0.2085 m during the five minute walk (280 m). The position error of the proposed algorithm with respect to the Vicon data is shown in Figure 15 and Table 1.

**Table 1:** Average position error of proposed algorithm and ZUPT with respect to Vicon data.

	ZUPT		Proposed Algorithm
	Right Foot	Left Foot	Waist
Average position error (m)	0.4934	0.6033	0.2085

**Figure 16:**
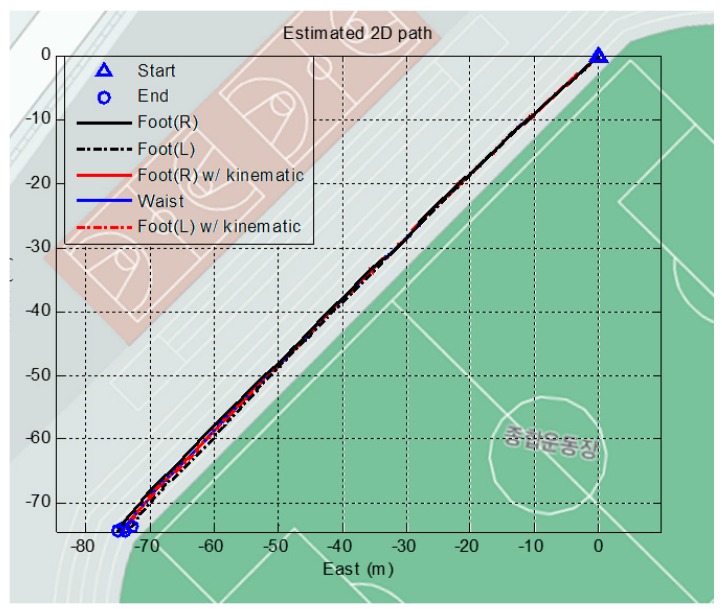
Result of straight line trajectories test (total distance: 106.06 m).

The algorithm is verified in an outdoor environment as well. The next test consists of two types of trajectories on the outdoor field: a straight line and a closed loop. First, the subjects are asked to walk along a straight line on the ground. This test verifies the performance of the proposed algorithm when no cornering is involved. The ZUPT updates the attitude of the foot-mounted sensor using only the gyro. Consequently, the yaw angle is observable only when cornering occurs. However, the proposed algorithm uses a magnetometer for the yaw angle, resulting in observations regardless of the course. Figure 16 shows the result of the straight line trajectory on a Google map. The blue line shows the position of the waist using the proposed algorithm, and red line shows the position of the feet, which is updated using the measured position of the waist. The walk of roughly 106 m straight line results in the distinctive paths depending on the algorithms. The two end points deviate over 2 m from each other.

The heading and position of the waist are correctly acquired and overlap with the Google map. The average total walking distance error of all the subjects is 0.673 m, which corresponds to a 0.6% error. Although the most important performance parameter is position error, not all the real positions of the pedestrian are recorded in this test. Therefore, it can be justified based on the comparison of the satellite map to the true trajectory that the proposed algorithm yields a high level of heading performance.

**Figure 17:**
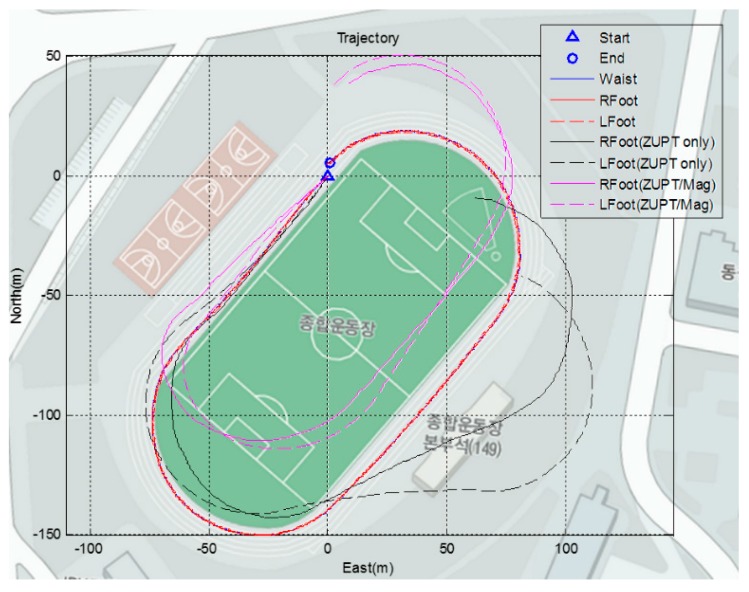
Result of playground trajectory test of each algorithm (total distance = 400 m).

Another outdoor test is conducted on a long distance closed loop. The subjects are asked to walk along the running track at a normal speed. The total distance of the trajectory is about 400 m. Figure 17 shows the results of the proposed algorithm in red and blue. The results of the PDR of the feet are represented in black, and the result of the ZUPT algorithm fusion with the heading of the waist, which is calculated using a magnetometer, is represented in magenta. It is noticeable from the figure that only the proposed algorithm yields trajectories close enough to the actual map. Furthermore, all the segment trajectories are placed near the waist trajectories, meaning that the positions of the segments are properly associated with physical coverage. The pure ZUPT algorithm applied to the foot yields curved trajectories due to the gyro bias. Using the yaw of the waist mounted sensor (magenta line), the ZUPT combined with corrected heading fails to estimate the actual human position, especially during cornering.

**Figure 18:**
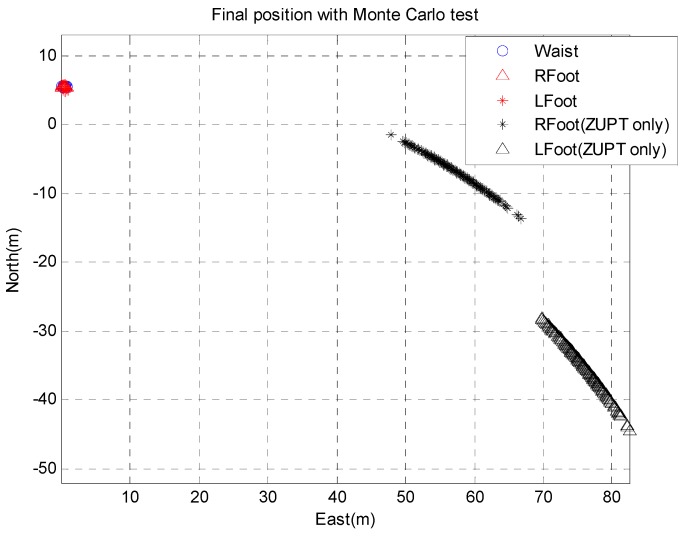
Final position of ZUPT algorithm and proposed algorithm for Monte Carlo simulation 500 times with random gyro drift error of 0.01°/s given.

The Monte Carlo test of 50 runs is conducted as another verification of the proposed algorithm. Random gyro bias (0.01°/s) is added to the experimental gyro data. The results of the Monte Carlo test are presented in Figure 18, which shows the final positions of each run, while the starting point is the origin for all runs. 

The final positions of the ZUPT algorithm are dispersed in a parabolic pattern because of the gyro bias. However, the proposed algorithm corrects the ZUPT algorithm and shows the exact location regardless of the gyro bias difference. The Return Position Error (RPE) of the PDR shows measurements of 57.7 m (left foot) and 83.2 m (right foot) and reveals that the primary source of error is the heading accuracy. In contrast, the proposed algorithm shows a RPE of 5.35 m (see Table 2). In consideration of the trajectory results, it is clear that the proposed algorithm is not just a mean of the two PDR systems, but a proper compensation method for the gyro bias and position error, in which the heading is corrected according to the magnetometer measurements.

**Table 2:** Average of RPE of each algorithm after 500 times of Monte Carlo test. Tested trajectory is 400 m of ground course.

	ZUPT		Proposed Algorithm		
	Right Foot	Left Foot	Right Foot	Left Foot	Waist
RPE(m)	57.3375	83.5120	5.4754	5.1073	5.5526

**Figure 19:**
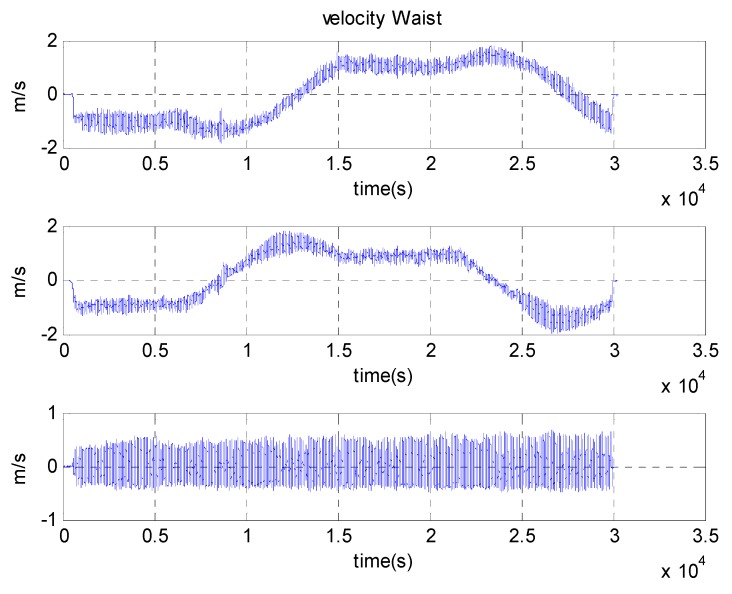
Results of estimated velocity of waist in NED frame during long distance trajectory tests.

**Figure 20:**
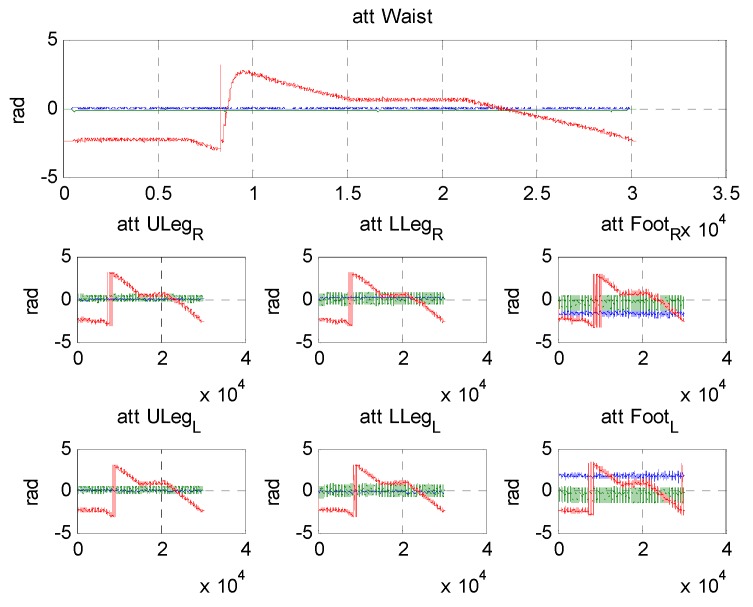
Results of estimated attitude of all segments during long distance trajectory (blue: roll, green: pitch, red: yaw). Attitude of right leg segments located at second row, and left leg segments located at third row.

Figure 19 and Figure 20 show the results of the estimated velocity of the waist and attitude of the segments during the 400 m-walk test. The velocity measurement, which is calculated from the kinematic model, correctly estimates the waist velocity. The true velocity of the pedestrian is not acquired in this test, and the accuracy of the results cannot be directly evaluated. However, comparing the magnitude of the velocity represented in NED frame to the Google map allows the filter to provide correctly estimated results. In the same way, the attitude of segment represented in Figure 20 shows that it is estimated correctly in physical meaning. The roll is stable near the origin, and the pitch waves periodically especially for the upper and lower legs. The yaw angle of the waist also nicely follows the heading of the waist, which contains the magnetometer information.

## 5. Conclusions

This study presented an enhanced method for finding heading and position of a pedestrian using a kinematic equation based on data from wearable sensor measurements. The IEZ algorithm-based PDR algorithm using the foot-mounted IMU was able to provide relatively accurate pedestrian positions. However, the heading of the pedestrian could not be solved using only the foot-mounted IMU. Moreover, fusing the step length of the IEZ with a heading of the waist-mounted IMU sensor was not a solution to the problem because the heading of the waist did not fully describe the direction of the feet. Consequently, the position of the pedestrian was incorrect. The overarching concept of the proposed method is that, by attaching wearable sensors to the lower parts of the body, estimation of the real position of the pedestrian can be obtained. The position and velocity of the waist measurements can be calculated through the kinematic relation update that estimates the drift of the waist sensor. The estimated waist position is inversely updated according to the lower limb, and the positions of the other segments are corrected based on this physical connection. Measurement of the foot position is transmitted to the IEZ model and allows the filter to observe the yaw direction. The proposed algorithm proved its ability to provide all of the constraint positions of the lower body segments and usefulness in tracking motions in a given area.

Several validation tests were conducted in both indoor and outdoor environments. The indoor performance was compared to a vision-based reference system. The position of the feet using the IEZ algorithm showed wrong directions and therefore produced deviations from the correct trajectory. Otherwise, the proposed system was able to obtain the accurate positions of all segments. Combining the foot position with the yaw direction of the waist mounted sensor, which was estimated using the magnetometer, could not fully describe the motion of the feet. The outdoor performance was justified by a comparison with a Google map. The results showed that the error is as low as 5 m in a 400 m walk, and the trajectories of the feet closely followed that of the waist.
